# Two Ends of the Leash: Relations Between Personality of Shelter Volunteers and On-leash Walking Behavior With Shelter Dogs

**DOI:** 10.3389/fpsyg.2021.619715

**Published:** 2021-04-14

**Authors:** Hao-Yu Shih, Mandy B. A. Paterson, Fillipe Georgiou, Leander Mitchell, Nancy A. Pachana, Clive J. C. Phillips

**Affiliations:** ^1^School of Veterinary Science, University of Queensland, Gatton, QLD, Australia; ^2^Royal Society for the Prevention of Cruelty to Animals Queensland, Brisbane, QLD, Australia; ^3^School of Mathematical and Physical Sciences, University of Newcastle, Callaghan, NSW, Australia; ^4^School of Psychology, University of Queensland, St Lucia, QLD, Australia; ^5^Curtin University Sustainable Policy (CUSP) Institute, Bentley, WA, Australia

**Keywords:** personality, dog, leash tension, dog-waking, shelter, human behavior, canine behavior, human-dog interaction

## Abstract

Human personality influences the way people interact with dogs. This study investigated the associations between the personality of animal shelter volunteers and behavior during on-leash walks with shelter dogs. Video recording and a canine leash tension meter were used to monitor the on-leash walking. Personality was measured in five dimensions (neurotic, extroverted, open, agreeable and conscientious) with the NEO Five-Factor Inventory (NEO-FFI). Neurotic volunteers pulled the leash harder and tended to interact with dogs using more body language; dogs being walked by neurotic volunteers in turn displayed more lip-licking and body shaking and were more likely to be rated as well-behaved. Extroverted volunteers were associated with stronger maximal leash tension at both the human and dog ends of the leash, and they praised the dog more, often in a high pitched voice. These volunteers eliciting more tail-wagging and body shaking by the dog. Extroverted volunteers were also more tolerant of different dog behaviors. Volunteers with personalities characterized by “openness to experiences” were less likely to verbally attract the attention of dogs, praise dogs and talk to them in a high-pitched voice; however, dogs walked by these volunteers were more likely to pull on the leash, and engaged in more lip-licking but less sniffing. “Agreeable” volunteers liked to verbally attract the attention of the dogs and more commonly initiated hand gestures and physical contact, causing the dogs to pull less frequently; dogs in these dyads displayed more gazing and lip-licking behaviors. Conscientious volunteers were less likely to pull the leash and tended to have more physical contact with the dogs but did not favor verbal communication and did not use a high pitched voice.

## Introduction

Dogs (*Canis familiaris*) show high sociability toward humans due to domestication and artificial selection, which enables them to share close relationships with us (Wheat et al., 2019). There are ~4,759,700 dogs in Australia, and 3,555,000 Australian households (38.5%) own at least one dog (Animal Medicines Australia, 2016). In the U.S. and U.K., there are an estimated 77,000,000 (American Veterinary Medical Association, 2018) and 9,000,000 (Pet Food Manufacturers' Association, 2019) pet dogs, respectively. Therefore, a thorough understanding of human-dog interactions is warranted, particularly in the fields of interspecific communicative skills (Hare and Tomasello, 2005), play (Rooney et al., 2000), stress modulation (Bergamascoa et al., 2010) and the gender differences in human-dog interaction (Aliabadi et al., 2011).

Despite the comprehensive literature on human-dog interactions, limited research has focused on the influence of human personality (Kis et al., 2012). Human personality is widely accepted to be associated with our perceptions and behaviors (Tasa et al., 2010; Kis et al., 2012), including the way we interact with dogs (Wedl et al., 2010; Kis et al., 2012; Cimarelli et al., 2016, 2017). The “similarity-attraction hypothesis,” which proposes that we share similar personality traits, physical attractiveness, and attitudes with our partners, has also been used to describe the owner-dog partnership (Turcsán et al., 2012). For instance, neurotic owners are more likely to own anxious dogs (Turcsán et al., 2012), and owners who score higher on psychoticism on the Eysenck Personality Questionnaire tend to own a dog of an allegedly “aggressive” breed, such as German shepherd or Rottweiler (Wells and Hepper, 2012). However, other studies have shown that dogs of neurotic owners were usually confident and friendly, with a lower corticoid reactivity (Kotrschal et al., 2009; Schöberl et al., 2012). One study even found no associations between dog and human personality (Walker, 2014). It is likely that the most satisfied owner-dog relationships occur between active owners and similarly active dogs (Curb et al., 2013).

It is expected that human personality influences the way people interact with dogs. Open and neurotic owners tend to use more gestural and verbal cues when commanding their dogs, while extroverted owners praise their dogs more often (Kis et al., 2012). Owners scoring high for openness score low for owner control (the tendency to use commands) (Cimarelli et al., 2017) but high on owner warmth (enthusiasm and prone to communicating with rather than commanding their dogs) (Cimarelli et al., 2016). Working dog handlers with high ratings for agreeableness tended not to clash with dogs and were less inclined to verbally correct them during the training (Payne et al., 2015). Owners scoring high for conscientiousness score lower on owner social support (inclined toward praising and petting) (Cimarelli et al., 2017). As for the dogs, they take longer to obey a command if owners score high on neuroticism (Kis et al., 2012). Dogs of owners scoring low on agreeableness, emotional stability, extraversion and conscientiousness are more likely to display owner-directed aggression, stranger-directed fear, and/or house soiling (Dodman et al., 2018; Gobbo and Zupan, 2020).

The majority of studies related to human personality and the human-dog dyad used owners and pet dogs (Kis et al., 2012; Turcsán et al., 2012; Cimarelli et al., 2017), thereby ensuring a relatively long-term relationship with a strong bond in most relationships. Similar studies have not been conducted in animal shelters (Walker, 2014), where dogs generally have had a shorter relationship and a weaker bond with shelter volunteers/personnel. Additionally, most of the studies were conducted when dogs were off leash (Kotrschal et al., 2009; Kis et al., 2012). Compulsory dog leash laws have been implemented worldwide to protect wildlife (Bowes et al., 2017), reduce disease transmission (Day et al., 2012), prevent dog attacks and dog involvement in traffic accidents on roads (Thompson, 1997; Klainbart et al., 2018). Therefore, training dogs to walk loosely on a leash is becoming increasingly important, because a tense leash may compress the trachea and damage their eyes through increased intraocular pressure (Pauli et al., 2006).

Equine rein tension meters have been developed to measure the force exerted on the reins during horse riding (Warren-Smith et al., 2007; Hawson et al., 2014). In our study, a similar concept was adopted to develop a custom-made leash tension meter that measures the leash tension when a dog is walked on a leash. This device also differentiates the direction (dog vs. human handler) of the pulling during the walk, using a three-axis accelerometer (Dewhirst et al., 2017; Shih et al., 2020a).

This study was part of a larger research project exploring the on-leash interaction between shelter dogs and volunteers. The project found that compared to men, women tended to engage in the interaction using more verbal commands, and language typically used with babies, while men were more inclined to have physical contact with dogs (Shih et al., 2020b). Dogs showed more stress-related behaviors (e.g., less time holding the tail in the high position, more frequent gazing and lip-licking) when interacting with men than women (Shih et al., 2020b). Other dog-related characteristics also affect the interaction, such as young (Shih et al., 2020a) and male (Shih et al., 2020b) dogs doing more pulling, more socialized dogs displaying more positive behavioral signals (tail in a high position and exploring the environment); thus volunteers utilized fewer verbal cues and body language when interacting with them (Shih et al., 2020c).

In this study, we focused on the relationships of human personality and the human-dog dyad when volunteers walked dogs on a leash. It was hypothesized that volunteers with higher scores on neuroticism would use more gestural and verbal cues (Kis et al., 2012) and pull harder on the leash, in order to have a better control over the dog (Kotrschal et al., 2009). In line with a previous study, volunteers scoring high on extraversion were expected to praise the dogs more (Kis et al., 2012), and those that were more open (Cimarelli et al., 2017) or agreeable (Payne et al., 2015) would pull the leash less frequently and allow dogs to explore without interfering with them by commanding or attempting to attract their attention. Frequency of dog pulling might increase in volunteers with more open personalities if they allowed the dogs to explore freely when walking (Cimarelli et al., 2017). Finally, agreeable volunteers may be more sensitive to the need of dogs and less inclined to compete with dogs, leading to less leash pulling (Payne et al., 2015).

## Materials and Methods

### Ethics Statement

This study was approved by the Human Research Ethics and Animal Ethics Committees (Approval numbers: 2018001570 and SVS/400/18, respectively), of The University of Queensland.

### Study Site

The research was conducted at the Royal Society for the Prevention of Cruelty to Animals, Queensland (RSPCA, QLD) shelter. Dogs were housed individually in rows of adjacent kennels (1.8 m wide × 1.2 m long × 3 m high) indoors and were able to make visual but not physical contact with each other across the central passage. Every enclosure was furnished with a metal crate, a raised mattress, a water bowl and enrichment (e.g., rubber toys or cardboard boxes). Each dog was walked twice daily, once in the morning from 08:00 to 10:00 h, and once in the afternoon from 14:00 to 16:00 h. The walk started from the kennel and ended with return to the kennel, taking about 15 min to complete. Moreover, dogs had plenty of time interacting with shelter volunteers, staff and visitors. These interactions provided the dogs with abundant social stimuli and enrichment. In this study we only recorded the interaction when dogs were on the designated pathway, with dogs walked uni-directionally, so as not to be confounded by turning around. The time on the designated pathway was ~5 min. The pathway ground had several sections with different coverings to provide tactile and olfactory enrichment for the dogs. The first 40% was covered with gravel, followed by 20% on a concrete section, then 5% on wooden boards, and finally the last 35% was covered with earth. Equipment and infrastructure provided added stimulation and play including two bridges, two dog jumps, some tennis balls and some hanging plastic milk bottles.

### Subjects

#### Dogs

This study investigated 370 walks from July to early October 2019, involving 111 shelter dogs and 74 volunteers. All participating dogs had to have been resident at the RSPCA, QLD, for at least a week to enable them to become accustomed to the living and walking areas. Dogs' walking behavior was categorized into four levels by RSPCA animal attendants who had been closely working with those dogs. Levels were assigned according to the ease of walking the dogs and was based on their performance during the daily walk. Level 1 dogs walked on a loose leash most of the time. Level 2 dogs pulled on the leash during the walk occasionally and had more undesirable behaviors than level 1 dogs. Level 3 dogs tended to pull on the leash fiercely due to excitement or timidity. Level 3+ dogs had severe behavioral issues, such as overt excitement or fearfulness; however, they were not believed to necessarily pull on the leash harder than level 3 dogs. Dogs with severe behavioral or medical issues that might affect the experiment were excluded from the study because of safety and welfare concerns. All included dogs had undergone an RSPCA behavioral assessment (Clay et al., 2019).

#### Volunteers

Volunteers were trained progressively in four stages, allowing them initially to walk level 1 dogs; at each learning stage they learnt how to walk the more challenging dogs. Volunteers could only walk level 1 dogs during their 1st month of volunteering and level 3+ dogs were allowed to be walked only by volunteers who were most experienced and had gone through a series of standardized training programs. Volunteers could only walk dogs that had the same or lower behavioral level than their training level. Dogs were assigned to volunteers by RSPCA staff for a daily walk based on each volunteer's training level.

#### Canine Leash Tension Meter

A custom-designed canine leash tension meter (sampling rate: 10 Hz; measuring range: 0–100 kg force; resolution: 100 g force) was commissioned for this project (RobacScience Australia) (Shih et al., 2020a). The device measured the force exerted on the leash and detected the direction of the pull (handler vs. dog). One end of the device had a metal handle to be held by the handler. The opposite end of the device had a stainless-steel eyebolt to allow a simple connection to a l.4 m long commercial dog leash (Rogz Snake Lead). A Windows® 10 personal computer program was written for data collection and real-time displays. Recorded data was processed using MATLAB® (MATLAB® and Statistics Toolbox Release 2018b, The MathWorks, Inc., Natick, Massachusetts, United States) (Shih et al., 2020a).

#### Measures

Participants completed a consent form for the research, and demographic and personality tests prior to participating in the study if they received the research information by email/poster, or after the experiment if they were approached by RSPCA staff and our researcher in person on the day. The personality test used for the study, the NEO Five-Factor Inventory, measures human personality in five factors: neuroticism, extraversion, openness, agreeableness and conscientiousness (McCrae and Costa, 2007). Neuroticism indicates a tendency to experience negative emotions, such as sadness, guilt, fear, and embarrassment. Extraversion represents a preference to be sociable and excited. Openness describes people's active imagination and their preference for variety. Agreeableness reflects individuals' orientations to people, displaying traits such as altruism, trusting, and wellness obtained from helping others. Conscientiousness includes a proclivity for impulse-control and self-discipline (Costa and McCrae, 1992).

#### Study Design

Volunteers who walked dogs were recruited via e-mail, poster, and direct recruitment by RSPCA staff and the lead researcher (H.-Y.S.). Prize draws for $20 RSPCA World for Pets vouchers were offered as incentives. Since there were very few level 1 dogs available during the research period, only volunteers with level 2 and above training levels were recruited. All participants had sufficient English proficiency to follow the research instructions and complete required documents. The research process was explained before the observational study and participants were informed on the research consent form that the overall study aim was to improve the interaction between volunteers and shelter dogs.

Dogs classified at the different levels were matched to volunteers of the right experience and training level by RSPCA staff. Dogs were walked on a designated pathway away from distractions at the shelter. Before every walk, the researcher held the leash tension meter vertically downward for 10 s without connecting it to the dog. The signals generated were later used to calibrate the recorded data using MATLAB®. The volunteer connected the leash to the dog's collar and harness in front of its chest via the leash tension meter. A laptop (Swift 3, Acer Inc., New Taipei City, Taiwan) was carried by the volunteer in a backpack for data collection, and a camera (GoPro Hero 7 Silver, GoPro®, California, United States) was mounted on the volunteer's head to record the interaction. Before walking, the volunteer was directed to pull the two ends of the device and hold the pull for 3 s by counting slowly “1, 2, 3.” This procedure was repeated three times, in order to synchronize the tension data with the video. During the walk, the volunteer was instructed not to touch the leash unless the dog became tangled. The researcher also recorded the walk using an i-Phone 7 (Apple Inc., California, United States) from 10 m behind. At the end of each walk, participants completed an exit questionnaire (Table 1) containing 13 questions about their perspective of the walk.

**Table 1:** Exit questionnaire for volunteers (*n* = 74) following walking dogs (*n* = 111) on a designated route at RSPCA Queensland, requiring them to rate each question on a 5-point scale from 1 (strongly disagree) to 5 (strongly agree) (Shih et al., 2020b).

1. The dog's behavior was good.
2. I could not handle the dog well.
3. I felt comfortable when interacting with the dog.
4. I was physically tense.
5. Overall, this is a good experience.
6. The interaction was challenging for me.
7. The dog did not understand me well.
8. I did not feel that I was helping the dog.
9. I felt supported by the dog.
10. I did not enjoy its company.
11. I would love to walk this dog again on another day.
12. I don't think this dog is suitable for a non-experienced adopter.
13. I think the dog is ready for adoption.

*Human satisfaction factor (Factor H): 2, 3, 4, 5, 6, 10, 11. Walker's “perception of dog” factor (Factor D): 1, 7, 8, 9, 12, 13. Factor loadings for the 13 items in the exit questionnaire can be found in Shih et al. (2020b). Statements 2, 4, 6, 7, 8, 10, and 12 required reverse scoring*.

### Data Analysis

#### Video Recordings of Dog and Human Behavior

Three hundred and sixty-eight (*n* = 368/370) videos were coded in their entirety with behavior observation software (Boris^©^, Friard and Gamba, 2016) using a continuous recording method. Two videos (*n* = 2/370) were unavailable due to technical problems. Canine behaviors (Table 2), human verbal cues (Table 3) and human body language (Table 4) were coded using ethograms developed based on previous research (Beerda et al., 1998; Palestrini et al., 2010; Kis et al., 2012; Cimarelli et al., 2016; Grainger et al., 2016; McGowan et al., 2018) and modified during practice sessions. Behaviors were coded as point or state events, these being the number of times an event was observed and the duration of the event, respectively. Videos were coded by the researcher who is a veterinarian and a certified dog trainer, trained by two senior Ph.D. students in the use of ethograms and the software. To blind the coder, video coding was completed prior to any analysis of human personality. Twenty percent of the videos were chosen at random and double-coded to check intra-rater reliability. The average Cohen's Kappa was 0.89.

**Table 2:** Ethogram of canine behavior.

**Behavior**	**Description**	**Behavior type**	**References**
Track	Dog moves along the ground with head lowered, using nose to follow a scent	State event	Grainger et al., 2016
Sniff	Dog orientates nose to within 5 cm of an object, wall or ground to explore or to express stress or appeasement	State event	Grainger et al., 2016
Eliminate-mark	Dog defecates or urinates in sitting, squatting or standing position	Point event	Palestrini et al., 2010
Shake	Dog shakes its body or head	Point event	
Pant	Dog keeps its mouth wide open and breathes vigorously	State event	Grainger et al., 2016
Gaze	Dog looks toward the handler	Point event	Grainger et al., 2016
Lip-lick	Part of tongue is shown and moved along the upper lip or snout	Point event	Grainger et al., 2016
Tail wag	Tail is moving from side to side	State event	McGowan et al., 2018
Tail high	Tail is held stiffly and upright, either curled over the back or straight	State event	Beerda et al., 1998

*Point event: the number of times the event was observed. State event: the duration of the observed event*.

**Table 3:** Ethogram of human verbal cues.

**Behavior**	**Description**	**Behavior type**	**References**
Sit	Volunteer asks the dog to sit.	Point event	
Command	Volunteer talks to the dog with an utterance containing a single command (e.g., “Stay!” “Come!” “Let's go!”)	Point event	Kis et al., 2012
Attention seeking	Volunteer tries to get the attention of the dog and calls the dog by its name and/or the utterance of “Look!,” and/or clicking the tongue (“tze tze” sound)	Point event	Kis et al., 2012
High-pitched voice	Volunteer talks to the dog with high pitched voice or with baby-talk expressions	Point event	McGowan et al., 2018
Praise	Volunteer talks to the dog with a positive utterance (e.g., “Great!” “Well done!” “Good dog!”)	Point event	Kis et al., 2012; McGowan et al., 2018
Negative verbal cue	Volunteer talks to the dog with a negative utterance (e.g., “No!” “Bad dog!” “Don't …” “Stop chewing the lead” “Let the lead (it) go”)	Point event	
Communication	Volunteer tries to communicate with the dog or to ask the dog some questions. (e.g., “Which way do you want to go?” “What are you sniffing at?” “Do you want to fetch?” “Do you want to drink?”)	Point event	Cimarelli et al., 2016

*Point event: the number of times the event was observed. State event: the duration of the observed event*.

**Table 4:** Ethogram of human body language.

**Behavior**	**Description**	**Behavior type**	**References**
Gestural	Volunteers displays voluntary hand movement directed toward the dog (e.g., referential point, patting his/her own thigh, luring the dog with a hand or food)	Point event	Kis et al., 2012; McGowan et al., 2018
Physical contacts	Physical contacts initiated by the volunteer. Including contacts when treats were given	Point event	
Food reward	Food is given to the dog	Point event	

*Point event: the number of times the event was observed*.

#### Leash Tension Analysis

Thirty-one (*n* = 31/370) leash tension files were lost, which we believe to be because the metal handle and case used to contain the components blocked the signal in a certain orientation. This left 339 (*n* = 339/370) files for analysis (Shih et al., 2020a).

Leash tension and pulling directions were calculated using MATLAB® (MATLAB ®and Statistics Toolbox Release 2018b, The MathWorks, Inc., Natick, Massachusetts, United States). The start and end of each file were determined by matching the timestamps of video and the leash tension file and also by matching three signal peaks at the beginning of the walk with the three repeated “1, 2, 3” verbal cues counted by the volunteer. Data were interpolated in order to make the sample times evenly spaced. Tension was tarred by deducting the minimal value, which visually equaled the baseline value when the device was not connected to the dog, from all measured values. Peak and average tensions over the walk were calculated (Shih et al., 2020a).

A “pull event” was defined as a sharp peak of tension greater than the baseline tension, which corresponded to a sudden burst of pulling initiated by either the dog, the handler or both at the same time. A peak-finding algorithm was used to determine when “pull events” occurred, with 0.1% of the body weight force set as the threshold. An event started when the filtered tension exceeded the threshold and ended when either the filtered tension returned to less than the threshold or the sign of the filtered tension gradient changed from negative to positive (indicating the start of a new pull event). Additionally, the directional signal of the accelerometer during the sample immediately prior to the start of a “pull event” was used to determine the pulling direction.

Net maximal tension (NT_max_), maximal tension by dog (DT_max_) and handler (HT_max_) were defined as the maximal tension throughout the walk, recorded for the dog and handler, respectively. Mean tension was calculated by averaging all tension peaks above the threshold. Net mean tension (NT_mean_), mean tension by dog (DT_mean_) and mean tension by handler (HT_mean_) were defined as the mean tension throughout the walk, recorded for the dog and handler, respectively. Dog pulling frequency (DPF) and handler pulling frequency (HPF) were calculated by dividing the number of pulling events caused by the dog and the handler, respectively, by the total walking time.

### Statistical Analysis

Statistical analysis was conducted using RStudio Version 1.2.1335 (R Core Team, 2019) with packages leaps (Miller, 2020), MASS (Venables and Ripley, 2002), car (Fox and Weisberg, 2019), carData (Fox et al., 2020), Matrix (Bates and Maechler, 2019), polycor (Fox, 2019), plyr (Wickham, 2011), psych (Revelle, 2020), ggpubr (Kassambara, 2020), and nlme (Pinheiro et al., 2020).

To describe the exit questionnaire, exploratory factor analysis was performed with 13 questions, which revealed 2 factors (Shih et al., 2020b). Negative question wording was deliberately used for one half of the questions; for these questions, reverse scores were used for the calculation of mean scores of the 2 factors. A human satisfaction factor (factor H, Cronbach's alpha = 0.88) indicated the handler's feelings about the walk. A higher factor H score indicated that the handler was more satisfied with the interaction. A walker's “perception of dog” factor (factor D, Cronbach's alpha = 0.83) represented the human's perception of the dog's behavior. A high factor D score indicated that the handler considered the dog well behaved, more supportive and being helped by the handler (Table 1).

The 370 interactions were not independent, as dogs were assigned to participants according to the training levels due to safety and welfare considerations. Also, dogs that had been staying in the shelter longer during the research period tended to be walked more often. Generalized linear mixed models were used for analysis to address the repeated measurements. To reduce the numbers of predictors, a bivariate generalized linear model was used to analyze each combination of outcome variables (leash tension, pulling frequency, dog and human behavior and exit questionnaire scores) and predictors (human and dog demographics, human personality, canine behavioral assessment). In the analysis of the exit questionnaire, apart from the above predictors, canine and human behaviors, HT_max_, and HT_mean_ were also entered as predictors. Along with these predictors, NT _max_ and NT _mean_ were included for the analyses of human and canine behaviors. Predictors with *p* < 0.2 (Bursac et al., 2008; Cecatto et al., 2015) in the bivariate generalized linear model and those that were logically expected to influence the outcome variables (human personality and canine behavioral assessment results), regardless of the *p*-value, were included in the generalized linear mixed model as fixed effects. Participants' ID number and dogs' ID number were entered as random effects. Regression analysis started with a full model, in which all candidate variables were defined as predictors of interest. To reduce the potential type I error, predictors with the highest *p*-values were then removed in a stepwise manner until the result of the model became consistent. In addition to assessing significance, the change in the Bayesian Information Criteria (BIC) was used to assess whether the model improved by entering or removing variables.

Outcome variables were manually transformed for statistical analyses to meet the following assumptions of generalized linear mixed models: (1) residual normality (assessed by observation of quantile–quantile plots), (2) normality of the random effects (assessed by observation of quantile–quantile plots), and (3) homogeneity of variance of residuals (confirmed with either Levene's Test or visual inspection of boxplots). The assumption of no collinearity between covariates was evaluated from variance inflation factors (VIF, ensuring that VIF<2) (Zuur et al., 2010).

This research was part of a larger research project that explored the behavioral interaction between shelter dogs and volunteers during walks. In this paper, emphasis is placed on how human personality influenced the behavioral interaction while shelter dogs were being walked on leash by volunteers. The effects of human gender (Shih et al., 2020b) and canine factors (Shih et al., 2020c) have been reported in previous articles. The effects of body size, body weight, age and the behavioral level of dogs were also reported (Shih et al., 2020a). Other human factors (e.g., working experience, volunteering time and frequency, history of dog ownership and educational level) will be reported separately.

## Results

### Demographics

This study involved 111 shelter dogs, including 58 (52.3%) females and 53 (47.7%) males, all gonadectomized (Shih et al., 2020b). Participants were 47 (63.5%) women, 26 (35.1%) men and 1 (1.4%) person self-nominating as the third gender (Shih et al., 2020b), with an average age of 28.26 (± 14.6) years. The mean scores of the five personality traits were 24.97 (± 8.80, neuroticism), 27.24 (± 7.77, extraversion), 29.59 (± 6.41, openness), 34.35 (± 5.97, agreeableness) and 30.54 (± 7.23, conscientiousness) (Table 5). Compared to the general population, the median score (but not the mean score) of neuroticism of participants is at the average-high level. Fourteen men (8 were high and 6 were very high) and 22 women (14 were high and 8 were very high) had neurotic scores higher than the average of the reported norms. Scores of other personality dimensions all fell within the average range (McCrae and Costa, 2007).

**Table 5:** Minimum, quartile 1 (Q1), median, mean (± SD), quartile 3 (Q3), and maximum scores or five personality traits.

**Personality**	**Minimum**	**Q1**	**Median**	**Mean (± SD)**	**Q3**	**Maximum**
Neuroticism	3.00	18.25	26.00	24.97 (± 8.80)	30.75	43.00
Extraversion	12.00	21.00	26.50	27.24 (± 7.77)	31.00	43.00
Openness	15.00	25.00	29.50	29.59 (± 6.41)	34.00	46.00
Agreeableness	21.00	32.00	35.00	34.35 (± 5.97)	39.00	48.00
Conscientiousness	12.00	25.00	30.50	30.54 (± 7.23)	35.75	47.00

### Human Personality and Leash Tension

Neuroticism was positively correlated with maximal net tension (*p* = 0.003), maximal tension created by the dog (*p* = 0.014) and maximal (*p* = 0.0293) and mean (*p* = 0.0425) tension created by the human. Extraversion was positively correlated with maximal net tension (*p* = 0.036), maximal tension created by the dog (*p* = 0.021) and maximal (*p* = 0.041) tension created by the human. Openness was positively correlated (*p* = 0.0031), and agreeableness negatively correlated (*p* = 0.044) with dog pulling frequency. Conscientiousness was negatively correlated with human's pulling frequency (*p* = 0.039) (Table 6).

**Table 6:** Generalized linear mixed model of the effect of human personality on leash tension and pulling frequency.

**Personality**	**Log_10_NT_max_**	**Log_10_NT_mean_**	**Log_10_DT_max_**	**Log_10_DT_mean_**	**Log_10_DPF^1^**	**Log_10_HT_max_**	**Log_10_HT_mean_**	**Log_10_HPF^a^**
Neuroticism	β 0.01	β 0.0052	β 0.009	β 0.0046	β −0.0016	β 0.0081	β 0.0052	β −0.00075
	SE 0.0034	SE 0.0032	SE 0.0037	SE 0.0028	SE 0.0054	SE 0.0037	SE 0.0026	SE 0.0049
	*p* 0.003**	*p* 0.11	*p* 0.014*	*p* 0.098	*p* 0.76	*p* 0.029*	*p* 0.043*	*p* 0.88
Extraversion	β 0.0074	β 0.004	β 0.0094	β 0.0044	β 0.0038	β 0.0077	β 0.0048	β 0.0058
	SE 0.0035	SE 0.0035	SE 0.0041	SE 0.0029	SE 0.0059	SE 0.0038	SE 0.0027	SE 0.0053
	*p* 0.036*	*p* 0.26	*p* 0.021*	*p* 0.13	*p* 0.52	*p* 0.041*	*p* 0.074	*p* 0.27
Openness	β 0.0025	β −0.0026	β 0.0033	β −0.0048	β 0.018	β 0.0023	β −0.0022	β 0.01
	SE 0.0038	SE 0.0036	SE 0.0041	SE 0.003	SE 0.0061	SE 0.0041	SE 0.0029	SE 0.0055
	*p* 0.51	*p* 0.47	*p* 0.43	*p* 0.11	*p* 0.0031**	*p* 0.58	*p* 0.46	*p* 0.061
Agreeableness	β −0.0063	β −0.0054	β −0.0072	β −0.0049	β −0.014	β 0.00075	β −0.0028	β −0.0067
	SE 0.0043	SE 0.0039	SE 0.0046	SE 0.0034	SE 0.0067	SE 0.0046	SE 0.0033	SE 0.0061
	*p* 0.14	*p* 0.17	*p* 0.12	*p* 0.15	*p* 0.044*	*p* 0.87	*p* 0.39	*p* 0.28
Conscientiousness	β 0.0049	β −0.0031	β 0.0045	β −0.0023	β 0.00096	β 0.0014	β −0.0023	β −0.013
	SE 0.0042	SE 0.004	SE 0.0047	SE 0.0034	SE 0.0067	SE 0.0046	SE 0.0031	SE 0.0061
	*p* 0.25	*p* 0.44	*p* 0.33	*p* 0.5	*p* 0.89	*p* 0.77	*p* 0.47	*p* 0.039*

*Tension and pulling frequency were analyzed in log_10_ transformation*.

*NT_max_, maximal net leash tension*.

*NT_mean_, mean net leash tension*.

*DT_max_, maximal leash tension caused by dog*.

*DT_mean_, mean leash tension caused by dog*.

*HT_max_, maximal leash tension caused by handler*.

*HT_mean_, mean leash tension caused by handler*.

*DPF, dog pulling frequency*.

*HPF, handler pulling frequency*.

a *Pulling frequency = (Numbers of pulls)/(walking duration). A pull was defined as a bout of force > 0.1% of the dog's body weight force*.

*β, regression coefficient*.

*SE, standard error of β*.

p, p-value of the model (*p < 0.05, **p < 0.01).

### Human Personality and Human Behavior

The extraversion score was positively correlated with the frequencies of praise (*p* = 0.027) and high-pitched voice (*p* < 0.001). The openness score was negatively correlated with the frequencies of total verbal cues (*p* = 0.0039), attention seeking (*p* < 0.001), praise (*p* < 0.001) and high-pitched voice (*p* = 0.016). The agreeableness score was positively correlated with the frequency of attention seeking (*p* = 0.04). Finally, the conscientiousness score was negatively correlated with frequencies of communication (*p* < 0.001) and high-pitched voice (*p* = 0.035) (Table 7).

**Table 7:** Generalized linear mixed model of the effect of human personality on human verbal cues during the walk.

**Personality**	**Total verbal cue (no./sec)^a^**	**Attention seeking (no./sec)^b^**	**Communication (no./sec)^b^**	**Negative verbal cue (no./sec)^b^**	**Praise (no./sec)^a^**	**High-pitched voice (no./sec)^a^**	**Command (no./sec)^a^**
Neuroticism	β −0.0013	β −0.00081	β −0.001	β −0.000095	β −0.0009	β 0.00033	β −0.00054
	SE 0.001	SE 0.00095	SE 0.00073	SE 0.00053	SE 0.0007	SE 0.00062	SE 0.0007
	*p* 0.18	*p* 0.4	*p* 0.17	*p* 0.86	*p* 0.2	*p* 0.59	*p* 0.44
Extraversion	β 0.00021	β −0.00075	β 0.0013	β 0.000011	β 0.002	β 0.0025	β −0.00054
	SE 0.0012	SE 0.00099	SE 0.00086	SE 0.00054	SE 0.00089	SE 0.0007	SE 0.00086
	*p* 0.87	*p* 0.45	*p* 0.14	*p* 0.98	*p* 0.027*	*p* < 0.001***	*p* 0.53
Openness	β −0.0031	β −0.0035	β 0.00061	β −0.00069	β −0.0029	β −0.0016	β −0.0012
	SE 0.0011	SE 0.001	SE 0.00075	SE 0.00056	SE 0.00076	SE 0.00065	SE 0.00072
	*p* 0.0039**	*p* < 0.001***	*p* 0.41	*p* 0.22	*p* < 0.001***	*p* 0.016*	*p* 0.092
Agreeableness	β 0.0012	β 0.0025	β −0.00096	β 0.0012	β −0.00083	β 0.0013	β 0.000071
	SE 0.0013	SE 0.0012	SE 0.00084	SE 0.00069	SE 0.00089	SE 0.00078	SE 0.00089
	*p* 0.37	*p* 0.04*	*p* 0.26	*p* 0.08	*p* 0.35	*p* 0.09	*p* 0.94
Conscientiousness	β −0.00054	β −0.00082	β −0.003	β 0.0004	β −0.00077	β −0.0016	β −0.00025
	SE 0.0012	SE 0.0012	SE 0.00088	SE 0.00064	SE 0.00089	SE 0.00075	SE 0.00081
	*p* 0.65	*p* 0.5	*p* < 0.001***	*p* 0.53	*p* 0.38	*p* 0.035*	*p* 0.76

*All verbal cues were analyzed with frequency (numbers of the event/total walking time)*.

a *Analyzed to the power of 0.5*.

b *Analyzed to the power of 0.4*.

*β, regression coefficient*.

*SE, standard error of β*.

p, p-value of the model (*p < 0.05, **p < 0.01, ***p < 0.001).

With respect to body language, volunteers who scored high on neuroticism (*p* = 0.0094), extraversion (*p* = 0.038) and agreeableness (*p* < 0.001) interacted with dogs using a higher frequency of total body language. Specifically, a high frequency of hand gestures was observed when the participant scored higher on neuroticism (*p* = 0.016) and agreeableness (*p* = 0.0083); a high frequency of physical contact was seen in people scoring high on neuroticism (*p* = 0.0078), agreeableness (*p* = 0.0025) and conscientiousness (*p* = 0.0091) (Table 8). Finally, there was no significant correlation between human personality and the success rate of asking dogs to sit.

**Table 8:** Generalized linear mixed model of the effect of human personality on human body languages during the walk.

**Personality**	**Total body language (no./sec)^a^**	**Food reward (no./sec)**	**Hand gesture (no./sec)^b^**	**Physical contact (no./sec)^a^**
Neuroticism	β 0.0036	β −0.0000088	β 0.0016	β 0.003
	SE 0.0014	SE 0.000043	SE 0.00067	SE 0.0011
	*p* 0.0094**	*p* 0.84	*p* 0.016*	*p* 0.0078**
Extraversion	β 0.0035	β −0.000026	β 0.0014	β 0.002
	SE 0.0017	SE 0.000051	SE 0.0009	SE 0.0014
	*p* 0.038*	*p* 0.6	*p* 0.13	*p* 0.17
Openness	β −0.00098	β −0.000055	β 0.00032	β −0.00041
	SE 0.0014	SE 0.000047	SE 0.00077	SE 0.0012
	*p* 0.5	*p* 0.24	*p* 0.67	*p* 0.72
Agreeableness	β 0.0066	β 0.000096	β 0.0025	β 0.0044
	SE 0.0018	SE 0.000052	SE 0.00092	SE 0.0014
	*p* < 0.001***	*p* 0.066	*p* 0.0083**	*p* 0.0025**
Conscientiousness	β 0.0023	β 0.000027	β 0.001	β 0.0036
	SE 0.0016	SE 0.000048	SE 0.00086	SE 0.0014
	*p* 0.16	*p* 0.58	*p* 0.25	*p* 0.0091**

*All body languages were analyzed with frequency (numbers of the event/total walking time)*.

a *Analyzed in power of 0.3*.

b *Analyzed in power of 0.4*.

*β, regression coefficient*.

*SE, standard error of β*.

p, p-value of the model (^*^p < 0.05, ^**^p < 0.01, ^***^p < 0.001).

### Human Personality, Canine Behavior, and Walking Experience

The score of neuroticism was positively correlated with the frequency of dogs' shaking (*p* < 0.001). A high percentage of time spent tail wagging (*p* = 0.047) and a high frequency of shaking (*p* = 0.0017) were observed in dogs while interacting with more extravert volunteers. When interacting with volunteers scoring high on openness, dogs presented a high frequency of lip-licking (*p* = 0.012) but lower percentage of time spent sniffing (*p* = 0.017). A high score for volunteers' agreeableness was associated with dogs with a high frequency of gaze (*p* = 0.0077) and lip-licking (*p* = 0.023) (Table 9). Finally, volunteers who scored high on neuroticism (*p* = 0.039) and extraversion (*p* = 0.0079) were more likely to rate higher on factor D (Table 10).

**Table 9:** Generalized linear mixed model of the effect of human personality on canine behavior during the walk.

**Personality**	**Track (%)**	**Tail high (%)^a^**	**Tail wag (%)^b^**	**Gaze (no./sec)^c^**	**Lip-lick (no./sec)^c^**	**Eliminate-mark (no./sec)^d^**	**Shake (no./sec)^e^**	**Pant (%)^f^**	**Sniff (%)^f^**
Neuroticism	β −0.00065	β 0.00028	β 0.002	β 0.0012	β 0.0015	β −0.000069	β 0.00019	β −0.0018	β 0.00056
	SE 0.00084	SE 0.0021	SE 0.0015	SE 0.00074	SE 0.00072	SE 0.00022	SE 0.000051	SE 0.0014	SE 0.00094
	*p* 0.44	*p* 0.89	*p* 0.19	*p* 0.11	*p* 0.04	*p* 0.76	*p* < 0.001***	*p* 0.2	*p* 0.55
Extraversion	β 0.00097	β 0.0015	β 0.0037	β 0.0014	β 0.0012	β −0.00026	β 0.00017	β −0.00023	β −0.00015
	SE 0.00087	SE 0.0022	SE 0.0019	SE 0.00079	SE 0.00092	SE 0.00022	SE 0.000052	SE 0.0016	SE 0.0011
	*p* 0.27	*p* 0.49	*p* 0.047*	*p* 0.088	*p* 0.21	*p* 0.23	*p* 0.0017**	*p* 0.88	*p* 0.89
Openness	β −0.0012	β 0.0028	β 0.0015	β 0.00048	β 0.0021	β 0.00028	β 0.000039	β 0.0017	β −0.0025
	SE 0.00094	SE 0.0023	SE 0.0018	SE 0.00083	SE 0.00083	SE 0.00023	SE 0.000057	SE 0.0014	SE 0.0011
	*p* 0.21	*p* 0.24	*p* 0.41	*p* 0.56	*p* 0.012*	*p* 0.24	*p* 0.5	*p* 0.24	*p* 0.017*
Agreeableness	β −0.0015	β 0.0018	β 0.0037	β 0.0026	β 0.0022	β −0.00027	β −0.000019	β −0.0021	β 0.0012
	SE 0.0011	SE 0.0027	SE 0.002	SE 0.00098	SE 0.00096	SE 0.00026	SE 0.000063	SE 0.0018	SE 0.0013
	*p* 0.18	*p* 0.5	*p* 0.058	*p* 0.0077**	*p* 0.023*	*p* 0.31	*p* 0.76	*p* 0.23	*p* 0.34
Conscientiousness	β −0.0017	β 0.0029	β 0.0012	β −0.00084	β 0.000017	β 0.00025	β 0.000043	β 0.00073	β −0.0021
	SE 0.001	SE 0.0026	SE 0.002	SE 0.00091	SE 0.00085	SE 0.0003	SE 0.000063	SE 0.0017	SE 0.0012
	*p* 0.11	*p* 0.28	*p* 0.54	*p* 0.36	*p* 0.98	*p* 0.4	*p* 0.49	*p* 0.66	*p* 0.086

*Track (%), tracking time (s)/total walking time (s) × 100%*.

*Tail high (%), tail high time (s)/total walking time (s) × 100%*.

*Tail wag (%), tail wagging time (s) / total walking time (s) × 100%*.

*Gaze (/sec), Numbers of gazes / time when the dog's head was visible in the Gopro video (s)*.

*Lip-lick (/sec), Numbers of lip-licks / time when the dog's head was visible in the Gopro video (s)*.

*Eliminate-mark (/sec), Numbers of eliminate-marks / total walking time (s)*.

*Shake (/sec), Numbers of shakes / total walking time (s)*.

*Pant (%), painting time (s) / time when the dog's head was visible in the Gopro video (s) × 100%*.

*Sniff (%): sniffing time (s) / total walking time (s) × 100%*.

a *Analyzed in power of 7*.

b *Analyzed in power of 0.3*.

c *Analyzed in power of 0.4*.

d *Analyzed in power of 0.6*.

e *Analyzed in power of 0.8*.

f *Analyzed in power of 0.5*.

*Wagging tail, shaking body and sniffing were not entered into the generalized linear mixed model because both predictors, dog and human gender, had high p-values in the bivariate regression models*.

*β, regression coefficient*.

*SE, standard error of β*.

p, p-value of the model (^*^p < 0.05, ^**^p < 0.01, ^***^p < 0.001).

**Table 10:** Generalized linear mixed model of the effect of human personality on volunteers' walking experience.

**Personality**	**Factor H^a^**	**Factor D**
Neuroticism	β 19088	β 0.01
	SE 34524	SE 0.005
	*p* 0.58	*p* 0.039*
Extraversion	β 15999	β 0.018
	SE 42102	SE 0.0069
	*p* 0.7	*p* 0.0079**
Openness	β −8759	β −0.0039
	SE 34602	SE 0.0057
	*p* 0.8	*p* 0.5
Agreeableness	β −8757	β 0.0015
	SE 42046	SE 0.0067
	*p* 0.84	*p* 0.82
Conscientiousness	β −45729	β −0.0018
	SE 41651	SE 0.0063
	*p* 0.27	*p* 0.78

a *Analyzed in power of 10*.

*β, regression coefficient*.

*SE, standard error of β*.

p, p-value of the model (*p < 0.05, **p < 0.01).

### Leash Tension, Human Behavior, and Walking Experience

Net maximal tension of the leash was positively correlated with the frequency of total verbal cues (*p* = 0.035) and commands (*p* < 0.001). However, net mean tension of the leash was negatively associated with the frequency of total verbal cues (*p* = 0.027), use of a high-pitched voice (*p* = 0.017) and commands (*p* = 0.0011) (Appendix Table 1).

The frequency of using negative verbal cues was negatively correlated with the Factor D score (*p* = 0.046) while the frequency of physical contact was positively related to the Factor H score (*p* = 0.038). The mean leash tension created by dog was negatively associated with the factor H (*p* = 0.0066) and factor D (*p* = 0.011) score (Appendix Table 2).

## Discussion

### Neuroticism

Volunteers scoring high on neuroticism pulled the leash harder during the walk, which may be related to the frequent body language used by more neurotic people (Kis et al., 2012). Additionally, mothers with increased neuroticism are more assertive and less adaptive, often using a more controlling or forceful style in discipline contexts (Clark et al., 2000). Similarly, owners scoring high on neuroticism tend to use more gestural and verbal commands when asking their dogs to sit (Kis et al., 2012). However, in our study, volunteers who scored high on neuroticism did not use more verbal cues during walks, although they did use more body language (e.g., hand gestures and physical contacts). These inconclusive outcomes may result from the different nature of the human-dog partnerships in this study. Dogs of owners high in neuroticism obeyed commands with a longer latency (Kotrschal et al., 2009; Kis et al., 2012). In another study regarding the personality effect on human-dog working tasks revealed no correlation between neuroticism and the human-dog dyad in working tasks (Hoummady et al., 2016). In our study, the success rate of asking the dog to sit was not associated with human personality, although volunteers were not specifically instructed to ask the dog to sit during the walk.

When walking with people scoring high on neuroticism, dogs licked their lips and shook their body more frequently, which may be a potential indicator of stress (Kogan et al., 2012). This assumption can be further supported by the high leash tension that neurotic volunteers exerted, and the high maximal tension dogs responded with. However, a previous study found that dogs with owners high in neuroticism were more confident and friendly (Kotrschal et al., 2009). Physiologically, dogs with owners scoring high in neuroticism often have a low cortisol level (Schöberl et al., 2012, 2017; Sundman et al., 2019). This indicates that dogs may be less stressed when interacting with neurotic owners because they tend to perceive their dogs as social support and stay in closer proximity with their dogs, and such intimacy may dampen the canine stress level (Schöberl et al., 2012, 2017; Sundman et al., 2019), which is supported by our finding that neuroticism is positively correlated with physical contact initiated by handlers. Therefore, the high incidence of lip-licking of dogs when interacting with more neurotic volunteers may be interpreted as appeasement signals, which are important components of greeting and peaceful intentions that occur commonly with reduced inter-individual distance (Firnkes et al., 2017). Moreover, appeasement signals are more commonly observed in mild but not overtly threatening situations and are believed to help manage stress (Firnkes et al., 2017). In shelters generally, dogs may be slightly uncertain but still enjoy the interaction with neurotic volunteers (Glenk et al., 2014).

### Extraversion

Volunteers' extraversion was positively correlated with the leash tension from the human, in line with the more assertive and disciplinary parental style observed in more extraverted mothers (Clark et al., 2000). In line with previous research, our results revealed that more extravert volunteers praised dogs more often (Kis et al., 2012) and their conversations were more likely to be at a higher pitch (Imhof, 2010), which in our study was exemplified by talking to dogs with an excited high-pitched voice. They also tended to communicate with dogs using more body language generally. Since extravert people often find social stimuli to be rewarding (Wu et al., 2014), all these behaviors may be interpreted as a mean of actively engaging in social interaction with dogs.

Dogs increased maximal leash tension and kept their tails high more often when walking with extravert volunteers, potentially because dogs were aroused by the excited human praise and high-pitched voice, and were more engaged in the interaction with volunteers (McGowan et al., 2018). However, dogs might be mildly tense and anxious at the same time by more frequently shaking their bodies (Harper, 2011; Meyer and Forkman, 2014).

### Openness

Owners with increased openness were reported to use more gestural and verbal cues when asking their dogs to sit (Kis et al., 2012). They also prefer communicating with, instead of commanding, their dogs (Cimarelli et al., 2016, 2017). However, in this study, volunteers scoring high on openness used fewer verbal cues, including fewer attention-seeking phrases, praises and a high-pitched voice, which again may be due to the short-term relationship between volunteers and shelter dogs. Our results agree with previous research about human personality and interpersonal interaction quality, that people with more openness tend to pay more attention but talk less (Berry and Hansen, 2000). Another possible explanation may be that open people respect the autonomy of dogs more (Booth-Butterfield and Sidelinger, 1997; Cimarelli et al., 2017), preferring not to control and command their dogs (Cimarelli et al., 2016, 2017). Although increased communication has not been observed in open volunteers (Cimarelli et al., 2016), they tended not to verbally interfere with the dogs' behaviors, such as by attracting their attention. This assumption is supported by the fact that when walking with more open volunteers, dogs pulled more frequently, and this was not as prominent at the human end.

More lip-licking was observed in dogs walked by more open volunteers, which may be considered as a similar outcome, an appeasement behavior, as interacting with more neurotic volunteers. However, since open volunteers verbally interacted with dogs less frequently, and dogs spent less time sniffing, an indicator of avoiding an uncomfortable conflict situation (Cohen, 2007), the high incidence of lip-licking was more likely to be a sign of mild discomfort (Harper, 2011) resulting from the frequent dog pulls when they were allowed to freely explore.

### Agreeableness

Agreeableness refers to the characteristic and tendency of compliance, cooperation, altruism (Costa et al., 1991) and being more responsive to social cues (Wu et al., 2014). Working dog handlers scoring high on agreeableness were reported to use lower rates of verbal corrections and preferred to cooperate with their dogs (Payne et al., 2015). Similarly, more agreeable volunteers tended not to clash with dogs, and in response, when interacting with more agreeable volunteers, dogs did not need to frequently struggle and pull on the leash. Also, people with increased agreeableness enjoy lively social interactions (Wu et al., 2014), which may explain why more agreeable volunteers tended to attract the attention of dogs both verbally (e.g., attention seekers) and physically (e.g., hand gestures and physical contacts).

### Conscientiousness

Conscientious mothers are more responsive to their children by being more sensitive to children needs, providing support and comfort and following their lead (Clark et al., 2000). Our result showed that handlers with a high score on conscientiousness pulled the leash less frequently. A possible explanation may be that, similarly, conscientious handlers sensitively captured and promptly responded to the dogs' signals. They respected autonomy of the dog and adjusted their own behavior to its current state or needs instead of attempting to correct the dog.

Cimarelli et al. found that owners scoring high on conscientiousness praise and pet their dogs less frequently (Cimarelli et al., 2017). However, our results revealed no significant correlation between volunteers' conscientiousness and verbal praise, and a positive association with physical contact. Such disagreements may demonstrate the difference in interspecific relationships between owners and pet dogs vs. volunteers and shelter dogs. Important components of conscientiousness refer to individuals' level of self-control and is related to competent communication and effectively evaluating responses to interpersonal problems (Hullman et al., 2010). Therefore, more conscientious volunteers might have perceived verbal communications as a less effective approach in human-dog interactions and preferred using non-verbal communications, such as physical contact (Mills, 2005). This is supported by previous research about interpersonal communication that the positive link between conscientiousness and communication might primarily be through writing but not verbal communication (Macht and Nembhard, 2015). Finally, a high voice and less emotional stability has been associated with decreased conscientiousness (Imhof, 2010), which explains why more conscientious volunteers were less inclined to talk to shelter dogs with an excited high-pitched voice.

### Personality and Experience

Although extravert owners are more likely to return dogs to the shelter post-adoption (Walker, 2014), owners scoring high on extroversion mainly consider their dog as a companion for shared activities while owners who score highly in neuroticism view their dogs as social support and spend more time with them (Kotrschal et al., 2009). Similarly, we found that volunteers who scored highly on neuroticism and extraversion were both likely to feel supported by the dogs and liked to engage in more shared activities. Also, they were more likely to report that the dogs they interacted with were well-behaved. In a cat study, high owner agreeableness was associated with a high level of satisfaction with their cats (Finka et al., 2019). However, such a result was not found in our research, possibly due to the species difference.

### Leash Tension, Human Behavior, and Experience

Volunteers talked to dogs more frequently, using more commands, when the net maximal tension was higher, probably because there was greater need for verbal commands when there were strong and sudden pulls. However, the opposite results were observed with fewer high-pitched voices and commands when the net mean tension was higher. In the scenario of higher mean tension, dogs were generally more determined and pulling, resulting in a lack of interaction between dogs and handlers.

Volunteers perceived the dog as less obedient and less supportive when they more frequently corrected the dog with negative verbal cues. However, volunteers were more satisfied with the interaction when there was more frequent physical contact between the dog and the human (Protopopova and Wynne, 2014). Finally, volunteers were less satisfied with the interaction and perceived the dog as less obedient and less supportive when the dog was causing a higher mean tension on the leash.

A limitation of this study was that dogs were not randomly matched with participants. Due to concerns for animal welfare and human safety, dogs were assigned to participants based on canine behavior and participants' experiences. Also, the cultural backgrounds of participated volunteers were unclear, which might potentially influence their interaction with dogs (Hood, 1998). Nevertheless, this article suggests that human personality can affect the human-dog interaction when walking on a leash.

## Conclusions

This research provides a qualitative description of associations between human personality and the behavioral dyads that exist between volunteers and shelter dogs when walking on a leash. Neurotic volunteers pulled the leash harder during the walk and tended to interact with dogs using more body language; dogs in turn displayed more lip-licking and body shaking. Extroverted volunteers were associated with stronger maximal leash tension at both the human and dog ends, and they praised the dog more, often accompanied with a high-pitched voice. This elicited more tail-wagging and body shaking by the dog. Open volunteers were less likely to verbally attract the attention of dogs, praise the dogs and talk to them with a high-pitched voice; however, dogs were more likely to pull on the leash, accompanied by more lip-licking but less sniffing. Agreeable volunteers liked to verbally attract the attention of dogs and initiate more hand gestures and physical contact, causing the dogs to pull less frequently, often coupled with more gazing and lip-licking behaviors during the walk. Conscientious volunteers were less likely to pull the leash and tended to have more physical contact with dogs, but they did not prefer verbal communication and a high-pitched voice.

In this study, dogs were more likely to be rated as well-behaved when walking with neurotic and extroverted volunteers. A more satisfactory human-dog relationship was detected if the owner and dog shared a similar activity level (Curb et al., 2013), while a mismatch between owner and dog personality increased the likelihood of dogs developing behavior issues (Dubé et al., 2020). Our study might contribute to a better matching of volunteers and dogs in animal shelters to improve the working experience and the welfare of dogs. Also, it may be used to better pair potential owners and dogs for more satisfying partnerships.

## Data Availability Statement

The raw data supporting the conclusions of this article will be made available by the authors, without undue reservation.

## Ethics Statement

The studies involving human participants were reviewed and approved by University of Queensland Science, Low & Negligible Risk Ethics Sub-Committee. Written informed consent to participate in this study was provided by the participants' legal guardian/next of kin. The animal study was reviewed and approved by Animal Ethics Unit, Office of Research Ethics, The University of Queensland.

## Author Contributions

H-YS, CP, MP, NP, LM, and FG: conceptualization. H-YS, CP, FG, MP, NP, and LM: methodology. FG and H-YS: software and writing—original draft preparation. H-YS, FG, and CP: validation, formal analysis, and data curation. H-YS: investigation. MP and CP: resources. H-YS, CP, MP, NP, and LM: writing—review and editing. H-YS and CP: visualization and funding acquisition. CP, MP, NP, and LM: supervision. H-YS, CP, and MP: project administration. All authors contributed to the article and approved the submitted version.

## Conflict of Interest

MP was employed as the principal scientist by RSPCA, QLD. The remaining authors declare that the research was conducted in the absence of any commercial or financial relationships that could be construed as a potential conflict of interest.
