# Supplementary material for: Two Ends of the Leash: Relations Between Personality of Shelter Volunteers and On-leash Walking Behavior With Shelter Dogs
Source: Front Psychol. 2021 Apr 14;12:619715. doi: 10.3389/fpsyg.2021.619715 (PMC8079626; doi:10.3389/fpsyg.2021.619715)
Supplement: Supplementary file 1 [file Table_1.docx]

Supplementary Material

# Supplementary Tables

**Appendix Table 1. Generalized linear mixed model of the effects of net maximal tension (NT_max_) and net mean tension (NT_mean_) on human verbal cues. All verbal cues were analysed with frequency (numbers of the event/total walking time).**

|  | Total verbal cues (no./sec)^a^ | Attention seeking (no./sec)^b^ | Communication (no./sec)^b^ | Negative verbal cue (no./sec)^b^ | Praise (no./sec)^a^ | High-pitched voice (no./sec)^a^ | Command (no./sec)^a^ |
| --- | --- | --- | --- | --- | --- | --- | --- |
| NT _max_ | *β* 0.0089  SE 0.0042  *p* 0.035^*^ | -- | -- | *β* 0.0033  SE 0.0024  *p* 0.17 | -- | -- | *β* 0.011  SE 0.0029  *p* < 0.001^***^ |
| NT _mean_ | *β* -0.071  SE 0.032  *p* 0.027^*^ | *β* -0.018  SE 0.025  *p* 0.48 | -- | *β* -0.0095  SE 0.018  *p* 0.59 | -- | *β* -0.04  SE 0.017  *p* 0.017^*^ | *β* -0.073  SE 0.022  *p* 0.0011^**^ |

1. Analyzed in power of 0.5.
2. Analyzed in power of 0.4.

*β*: regression coefficient

SE: standard error of *β*

*p*: *p* value of the model (*p*^*^ < 0.05, *p*^**^ < 0.01, *p*^***^ < 0.001)

**Appendix Table 2. Generalized linear mixed model of the effects of human behaviour, maximal tension by dog (DT_max_) and mean tension by dog (DT_mean_) on volunteers’ walking experience (factor H & factor D).**

|  | Factor H^a^ | Factor D |
| --- | --- | --- |
| Attention seeking (no./sec) | -- | *β* -3.06  SE 1.89  *p* 0.11 |
| Negative verbal cue (no./sec) | *β* -15842007  SE 39766630  *p* 0.69 | *β* -14.75  SE 7.35  *p* 0.046^*^ |
| Praise (no./sec) | *β* 8891984  SE 8823625  *p* 0.32 | *β* -0.26  SE 1.58  *p* 0.87 |
| Command (no./sec) | -- | *β* 2.065  SE 1.55  *p* 0.18 |
| Hand gesture (no./sec) | -- | *β* -4.97  SE 5.94  *p* 0.4 |
| Physical contact (no./sec) | *β* 77293346  SE 36890086  *p* 0.038^*^ | *β* 6.65  SE 6.18  *p* 0.28 |
| DT _max_ | *β* 86341  SE 177683  *p* 0.63 | *β* -0.016  SE 0.031  *p* 0.6 |
| DT _mean_ | *β* -1919702  SE 696659  *p* 0.0066^**^ | *β* -0.28  SE 0.11  *p* 0.011^*^ |

Human satisfaction factor (Factor H): A higher factor H score indicated that the handler was more satisfied with the interaction.

Dog behaviour factor (Factor D): A higher factor D score indicated that the handler considered the dog better behaved.

1. Analyzed in power of 10.

Total verbal cue, communication, high-pitched voice, total body language and food reward were not included in the model due to high *p* values.

*β*: regression coefficient

SE: standard error of *β*

*p*: *p* value of the model (*p*^*^ < 0.05, *p*^**^ < 0.01)
